# Productivity in medical education research: an examination of countries of origin

**DOI:** 10.1186/s12909-014-0243-8

**Published:** 2014-11-18

**Authors:** Asif Doja, Tanya Horsley, Margaret Sampson

**Affiliations:** Children’s Hospital of Eastern Ontario, 401 Smyth Road, Ottawa, ON K1H 8L1 Canada; Royal College of Physicians and Surgeons of Canada, Research Unit, 774 Echo Drive, Ottawa, K1S 5N8 Canada

**Keywords:** Bibliometrics, Academic productivity, Author networks

## Abstract

**Background:**

Productivity and countries of origin of publications within the field of medical education research have not been explored. Using bibliometric techniques we conducted an analysis of studies evaluating medical education interventions, examining the country where research originated as well as networks of authors within countries identified as ‘most productive’.

**Methods:**

PubMed was used to search for evaluative studies of medical education. We then examined relative productivity of countries with >100 publications in our sample (number of publications/number of medical schools in country). Author networks from the top 2 countries with the highest relative productivity were constructed.

**Results:**

6874 publications from 18,883 different authors were included. The countries with the highest relative publication productivity were Canada (37.1), Netherlands (28.3), New Zealand (27), the UK (23), and the U.S.A (17.1). Author collaboration networks differed in both numbers of authors and intensity of collaborations in the countries with highest relative productivity.

**Conclusions:**

In terms of the number of publications of evaluative studies in medical education, Canada

was the country with the highest relative productivity. Author networks allow for the identification of ongoing and potential new collaborations amongst authors.

**Electronic supplementary material:**

The online version of this article (doi:10.1186/s12909-014-0243-8) contains supplementary material, which is available to authorized users.

## Background

With an increasing emphasis on research and scholarship in the field of medical education, it becomes useful to explore the field of medical education research itself. An area of great interest from both an individual (i.e. researcher) and larger scale (i.e. country) perspective is through quantification measured by productivity. As the field develops and legitimizes globally, identifying the main drivers of research in medical education informs our understanding of whose “world view” is predominant within the medical literature and provides commentary for the generalizability of current science in medical education. Examining productivity of medical education research has not been examined critically, specifically as it pertains to how productive individual countries and authors are.

Previous research has provided initial insight into productivity albeit based on a restrictive dataset. Tutarel [1] examined the country of origin for articles published in two medical education journals, *Medical Education* and *Academic Medicine*, over five years (1995 to 2000). He found that authors from the USA and Canada were responsible for 95% of articles in *Academic Medicine* and that the UK, Australia, the USA, Canada and the Netherlands were responsible for 74% of the articles in *Medical Education*. In another study [2], he also found that the composition of editorial boards also lacked depth with a paucity of members from developing countries.

Similarly, Rotgans [3] performed a content analysis of 10,168 abstracts from the top six medical education journals as ranked by impact factor (*Academic Medicine, Advances in Health Sciences Education, Medical Education, Medical Teacher, Advances in Physiology Education, and Teaching and Learning in Medicine*) from 1988–2010. He did not examine the productivity of country of origin, per se, but rather listed the top 10 universities which generated the most publications.

While the above studies provide cursory insights into medical education research patterns, both are limited in that they examined only a narrow subset of medical education journals (likely introducing selection bias) as well as omitting non-medical education journals (e.g. general medicine and subspecialty journals), which are known to frequently publish medical education research [4].

A further limitation relates to the use of the absolute number of publications as a measure of productivity. Theoretically, a more accurate measure of a country’s publication productivity could meaningfully be derived by dividing the number of applicable publications (in this case medical education) by the number of medical education researchers within a given country.

Conceptually, this is not a difficulty notion to convey; however, operationally it is not easily derived. Medical education research is a discipline that includes individuals from many other ‘host disciplines’ (e.g. anthropologists, cognitive psychologists, sociologists), and because of this, it becomes almost impossible to fully estimate the number of academics (the denominator) specifically associated with medical education research geographically. However, since most medical education research is performed by individuals associated with a medical school, we hypothesized that the number of medical schools in a given country could serve as a reasonable proxy for the number of medical education researchers/academics. Thus, our goal was to examine the relative productivity, calculated as the number of medical education publications/number of medical schools in individual countries. To further our understanding of professional collaborations and patterns of networking we additionally sought to construct authorship network diagrams an illustration of the patterns of collaboration between authors in select countries.

## Methods

We searched PubMed for articles using the MeSH term “Education, Medical” or any of the more specific MeSH terms below it in the MeSH hierarchy [5-7]; *Continuing Medical Education, Graduate Medical Education, Undergraduate Medical Education, Clinical Clerkship, Internship and Residency and Teaching Rounds* to identify published evaluative studies indexed as medical education. We use the term *evaluative studies* to describe medical education articles describing one or more interventions and including at least one evaluative component. This allows for the inclusion of empirical studies ranging from randomized controlled trials to qualitative studies, but excludes commentaries, editorials, historical articles, and letters. Our group has previously developed a search methodology to identify such evaluative studies [4], and this was used in the current study.

One author (MS), a health sciences librarian, developed and executed the search through PubMed originally on January 26, 2012, and were updated to April 7, 2014 (Additional file 1). To obtain bibliometric data, the search was then repeated in GoPubMed, a service that analyzes aspects of a retrieved set of references including author, year, keyword, country, and journal frequencies. GoPubMed results were used as the source data for determining the number of publications by country of corresponding author. For comparison, articles indexed as medical education but not retrieved by the evaluative study filter were analyzed. Due to the limits imposed by GoPubMed, only the most recent 100,000 records can be analyzed. This corresponded to publications from 1974 to present.

GoPubMed co-authorship network diagrams were produced on Feb 29, 2012, for the most productive countries, by further limiting the search to corresponding authors providing that country as their location.

### Country characteristics

We examined relative productivity of the 15 countries with the most publications. The number of medical schools were established in a given country and then an average number of publications per school was estimated.

The main sources for the number of medical schools was the AVICENNA Directory (http://avicenna.ku.dk/database/medicine/) which is a directory of international medical schools maintained by the University of Copenhagen in collaboration with the World Health Organization and the World Federation for Medical Education. For the 15 countries included within our study, we also conducted a web search for medical schools in that country. When the number of medical schools apparent from the 2 sources disagreed, we used the higher number as it would provide the more conservative estimate of relative productivity.

Although our primary goal was to examine evaluative studies, for comparison, we also looked at the absolute numbers of publications from various countries for non-evaluative studies. Additionally for the top countries with the highest absolute number of publications, we determined the top five most productive cities.

We examined the productivity of countries using an independent database in order to validate our findings. Two databases frequently used for bibliometric analysis of the biomedical literature are Web of Science (Science Citation Index) and Scopus. Neither formally indexes articles by subject. The scientific literature in Scopus is drawn from the MEDLINE and Embase databases. As the indexed material in PubMed is drawn from MEDLINE, Scopus and PubMed are not independent sources. We therefore chose Web of Science as our validation source and we searched it for the overall term “Medical Education”. We compared the country rankings of these results with the results of a search for the index term “Education, Medical” in PubMed (through the GoPubMed interface).

## Results

**Figure 1:**
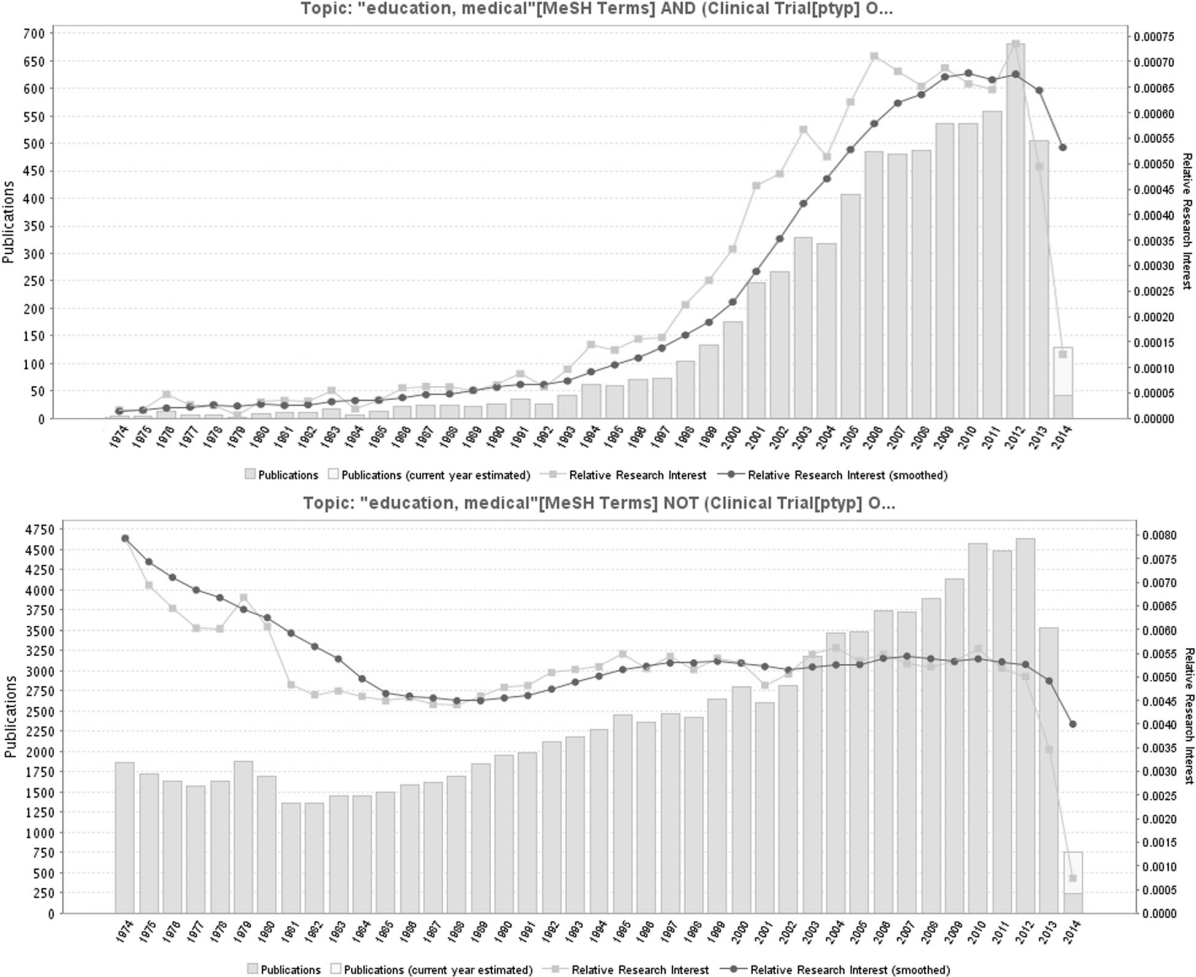
**Publications by year for evaluative and non-evaluative studies.** Data based on the first 100,000 record obtained in GoPubMed from 1974–2014. Data obtained on April 14, 2014.

The search for evaluative studies identified 6,896 documents, which was reduced to 6874 when limited to publications from 1974 onward. For the evaluative studies, the whole set was analyzed using GoPubMed, and counts were adjusted to remove the earliest 22 records. Across all years, 100,199 studies were indexed as medical education but not retrieved by our search for evaluative studies. This was limited to the newest 100,000, corresponding to a publication year of 1974 or more recent, for analysis by GoPubMed (see Figure 1).

For the evaluative studies, corresponding authors came from 108 different countries. The 15 most productive countries are shown in Table 1 with the top producing cities in these countries being listed in Table 2. Using absolute numbers of evaluative studies published, the USA, United Kingdom and Canada were most prolific (Table 1). however, when adjusting estimates using the number of medical schools per country, Canada, Netherlands and the New Zealand were the top 3 producing countries respectively, with New Zealand moving from 15^th^ position (unadjusted) to 3^d^ position (adjusted) (Table 1).

**Table 1:** **Productivity and relative productivity of countries for evaluative medical education studies**

**Rank**	**Country**	**N**	**% (of 6,896)**	**Medical schools**	**Ratio**	**Rank**
1	USA	2920	42.3	171	17.1	6
2	United Kingdom	852	12.4	37	23.0	4
3	Canada	630	9.1	17	37.1	1
4	Australia	278	4.0	25	11.1	8
5	Germany	232	3.4	42	5.5	11
6	Netherlands	226	3.3	8	28.3	2
7	Spain	92	1.3	38	2.4	16
8	France	92	1.3	43	2.1	19
9	Denmark	74	1.1	4	18.5	5
10	Italy	71	1.0	39	1.8	20
11	Sweden	70	1.0	6	11.7	7
12	Brazil	69	1.0	181	0.4	15
13	Japan	62	0.9	81	0.8	14
14	Switzerland	59	0.9	6	9.8	10
15	New Zealand	54	0.8	2	27.0	3

**Table 2:** **Productivity of 5 most productive cites for the countries with relatively highest productivity, plus USA, for evaluative medical education studies**

**Country**	**5 Most productive cities**	**Evaluative medical education studies**	**Total from 5 cities**	**National total**	**% of National production from 5 cities**
**Canada**	Toronto	217			
	Montréal	59			
	Hamilton	50			
	Vancouver	47			
	Calgary	43			
			416	630	66%
**Netherlands**	Amsterdam	58			
	Maastricht	46			
	Nijmegen	33			
	Groningen	21			
	Utrecht	20			
			178	226	79%
**New Zealand**	Auckland	20			
	Wellington	12			
	Dunedin	10			
	Christchurch	7			
	Hamilton	2			
			51	54	94%
**United Kingdom**	London	189			
	Manchester	43			
	Leeds	38			
	Birmingham	36			
	Bristol	35			
			341	852	40%
**Denmark**	Copenhagen	34			
	Århus	22			
	Odense	6			
	Sønderborg/Hvidovre/Vejle*	2			
			64	74	86%
**USA**	Boston	176			
	New York City	141			
	Philadelphia	127			
	Chicago	117			
	San Francisco	103	664	2920	23%

*These three cities are tied with 2 publications each.

When comparing the absolute numbers of publications from various countries for non-evaluative studies (Table 3), the top 6 countries with respect to absolute number of publications was the same for evaluative and non-evaluative studies.

**Table 3:** **Absolute number of publications per country for evaluative and non-evaluative studies**

**Non-evaluative records obtained (first 100,000)**		**Evaluative records obtained (6,814)**	
United States	27,173	United States	2,920
United Kingdom	5,559	United Kingdom	852
Canada	3,124	Canada	630
Germany	1,748	Australia	278
Australia	1,745	Germany	232
Netherlands	934	Netherlands	226
France	749	France	92
Japan	640	Spain	92
Sweden	543	Denmark	74
Italy	536	Italy	71
Spain	513	Sweden	70
India	476	Brazil	69
Switzerland	454	Japan	62
Brazil	435	Switzerland	59
New Zealand	408	New Zealand	54

Searching for the overall term “Medical Education” in Web of Science found 26,209 records. Comparisons of rankings derived from Web of Science and PubMed (using GoPubMed) are shown in Table 4. The order was the same for the first 6 countries, adding some confidence to our findings.

**Table 4:** **Comparison of number of studies indexed as “Medical Education” between PubMed (via GoPubMed) and Web of Science**

**Pubmed (via GoPubMed) (first 100,000)**		**Web of science (26,209)**	
United States	30,069	United States	11,902
United Kingdom	6,409	United Kingdom	2,069
Canada	3,751	Canada	2,048
Australia	2,023	Australia	1,058
Germany	1,980	Germany	882
Netherlands	1,158	Netherlands	651
France	840	Scotland	448
Japan	702	France	380
Sweden	613	India	300
Italy	607	Italy	223
Spain	603	China	221
Switzerland	513	Spain	216
India	511	Israel	213
Brazil	504	Sweden	203
New Zealand	462	Switzerland	202

**Figure 2:**
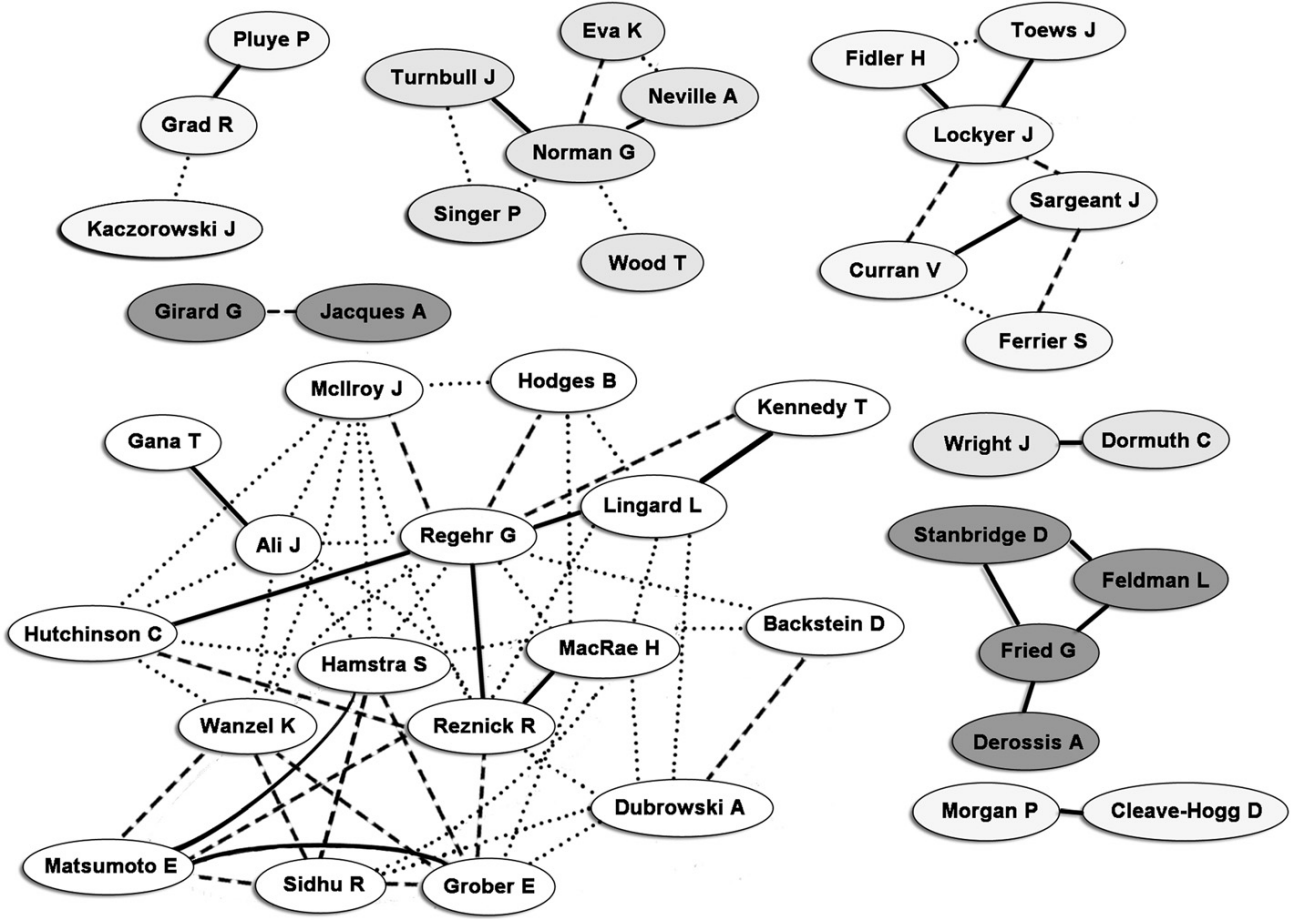
**Authorship collaborations for eligible papers from first authors from Canada.** Solid lines denote three or more co-publications, dashed lines denote two co-publications and dotted lined denote a single co-publication. Differential shading is used to more clearly distinguish between networks. Network representation is based on data from Feb 29, 2012.

**Figure 3:**
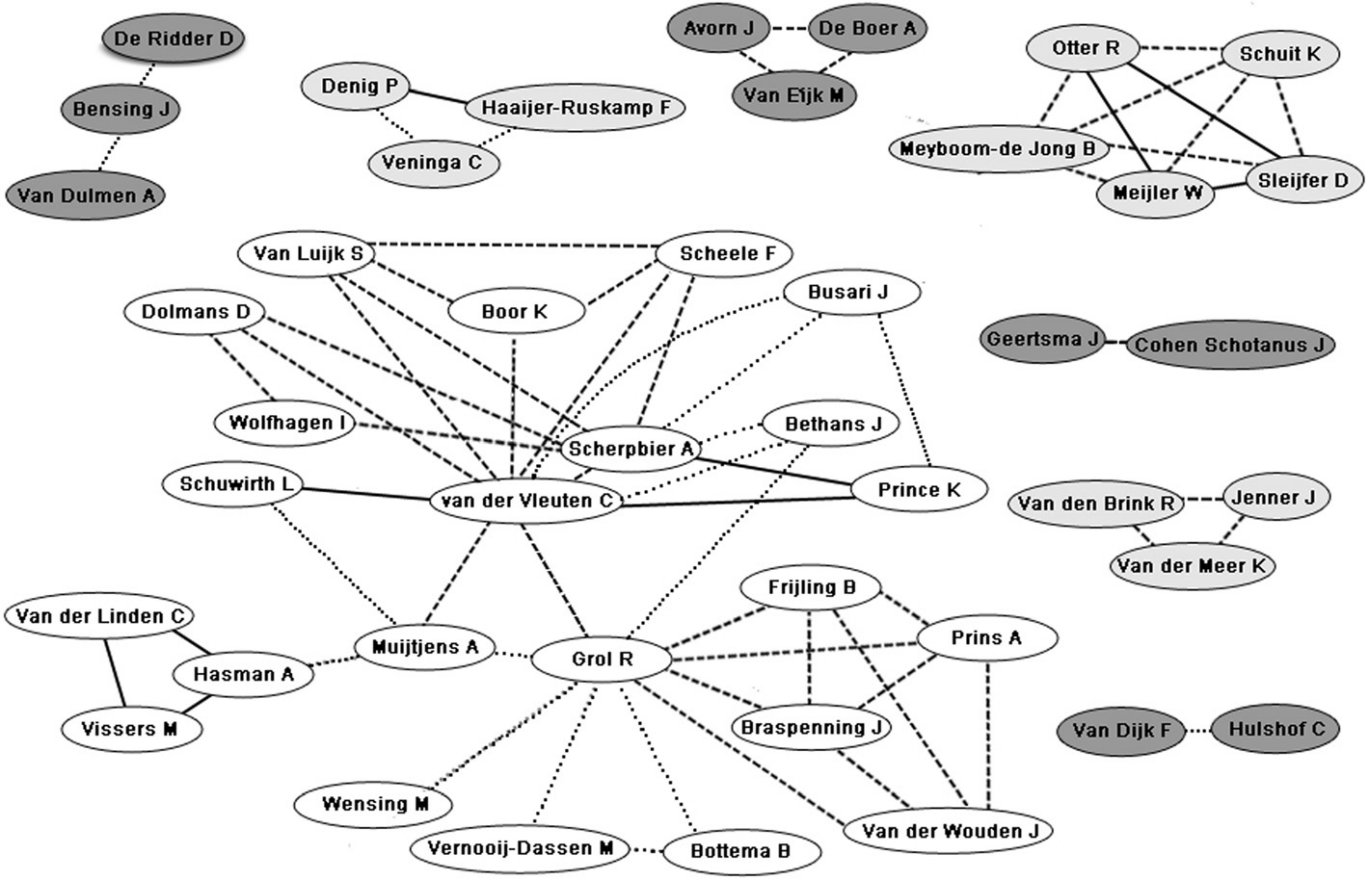
**Authorship collaborations for eligible papers from first authors from the Netherlands.** Solid lines denote three or more co-publications, dashed lines denote two co-publications and dotted lined denote a single co-publication. Differential shading is used to more clearly distinguish between networks. Network representation is based on data from Feb 29, 2012.

Author network diagrams for the 2 countries with the highest relative productivity are shown in Figures 2 and 3.

## Discussion

The purpose of the present study was to rigorously, and systematically identify the ‘most productive’ countries where medical education research originates as well as networks of authors within those countries.

As is demonstrated in Figure 1, there has been a significant increase in publications in medical education over the previous four decades. Both evaluative and non-evaluative studies have increased over time, but there is a distinct lack of evaluative studies prior to 2003. This dramatic increase may be resultant from the call for greater accountability in medical education research and the development of outcomes research methodologies in the early 2000s [8].

We found that the 5 countries with the most published evaluative studies in medical education were the USA, the United Kingdom, Canada, Australia and the Netherlands. Examination of cities the in the top countries provides further information regarding how this publications may be distributed in a given country. However when relative productivity was examined, the ranking of countries noticeably changed. Canada was ranked first with respect to relative productivity and New Zealand, while only having 37 publications, was calculated as the 3^d^ most productive. This indicates that despite only having 2 medical schools, within our analysis, New Zealand contributed significantly to medical education research literature.

There were no appreciable differences in the top 6 countries when comparing evaluative and non-evaluative studies. This may imply that for the most part, the same countries are producing empirical studies as well as the descriptive, narrative, “thought pieces” which are influencing the trajectory of the medical education literature.

Perhaps not surprisingly, those countries with the highest numbers of publications and relative productivity are can be considered high-income countries. These data could suggest that the ‘main drivers’ of medical education research predominate from a primarily Western viewpoint. However, this does not explain why a country such as the USA, which most would certainly consider a high-income nation, would be a relative ‘underperformer’. One explanation for this may have to do with the dual functions of medical schools– to train doctors and to perform research. Countries that have lower relative productivity may have medical schools more focussed on education as opposed to research.

Seemingly low productivity within medical education research in lower and middle income countries may also occur for a number of reasons. Medical education is now emerging as a legitimate field; infrastructure and process components may be less developed in low income countries and thus, likely less of priority within medical schools when compared to traditional biomedical research. Funding for medical education, research methods and metrics for faculty tenure and promotion may be additional barriers, since, in resource poor countries, funding for research in generally limited across any field.

Beyond the organic academic inquiry of ‘simply knowing’ the most productive countries and author networks within their borders, our findings could play an influential role to those looking for empiric ‘levers’ for setting, and perhaps negotiating, agendas for funding at institutional, local and national levels. In 2007, Todres, Stephenson and Jones examined the field of medical education and determined that research lacks methodological rigour and support from funding councils [9]. Research based in the United States echoes these findings suggesting funding is woefully inadequate for medical education research. Reed et al. [10] examined medical education publications from 2002–2003 and found that the majority of published medical education research was not formally funded, and the studies that did receive support were substantially underfunded. Carline [11] Examined funding patterns of North American medical education research within two dominant journals (Academic Medicine and Teaching and Learning in Medicine) and found that not only were few studies supported by external grant funds, most were of reduced ‘quality, rigour, and generalizability’. More recently, Ash and Weinstein [12] have called upon the need for outcomes based research and innovation in funding for medical education in the USA. Using our data could potentially open a dialogue for capacity building within countries where established author networks have not materialized -- and hence where productivity is comparatively low – and could be a catalyst for a identifying and supporting a cadre of scientists to mobilize local, institutional and national agendas.

While our findings have perhaps more obvious implications for ‘underperforming’ countries there are important implications for countries that emerged as ‘leaders’ in productivity. In Canada for example, funding for medical education research remains a challenge with few agencies ear-marking funding specific for health professions education research as compared with other specialty- or disease-specific areas of research. When considering the Canadian context, medical education researchers have have anecdotally been recognized as having international impact and recognition. Our findings provide evidence that Canada has strong record of medical education productivity and well developed author networks. Our findings may provide empiric evidence towards identifying Canada as a global leader in medical education research and a strong, evidence-informed negotiating platform for why more funding, in an organized and focused manner, is required for medical education research in Canada.

The authorship network diagram is a relatively new tool that can be utilized to describe existing and potentially new collaborations amongst authors. Several web services provide authorship network diagrams including GoPubMed, Scopus, Science Direct, and BioMedNet. Although network diagrams illustrate the breadth of collaboration practices by leading researchers, for example, to identify where strong collaborative networks advance an area of science, it should be noted that these diagrams are not indicative of the role played by each author within the collaborative network. For example, although the network diagrams we constructed encode the number of co-publications, they provide no insight into who is the first or senior author on the work. Thus, the diagrams do not provide critical insights into the power relationship of the authors in terms of primary vs. senior vs. collaborative position within a given paper. Additionally, PubMed, and so also GoPubMed, record only the country of the corresponding author. Therefore, although international collaborations are represented in the networks, they not apparent from in the diagrams. Despite these limitations, the networks demonstrate heterogeneous patterns when based on the country's high relative productivity, which suggests that there is no single, predictable, pattern for success. It may be important in the future to explore the nature of comparative collaborative networks, for example within medical education versus more established disciplines (e.g. within given a given clinical area such as HIV research) over time to better understand how networks have formed to develop and advance a scientific area and compare them to what is currently done in medical education.

There are several limitations to our study. We used the number of publications and relative productivity based on the number of medical schools to determine the productivity of countries. A more correct measure would be to use the number of full time (or full time equivalent) medical education researchers per country. However, since there is no established database of medical education researchers, this type of figure would be extremely difficult to obtain.

Other measures of academic productivity attempt to quantify the apparent impact of the work by considering how often the work has been cited. The journal impact factor [13] looks at the prestige of journals the articles are published in, based on the average number of citations per articles, but does not address the impact of the specific articles. Alternatively the *h*-index (Hirsch Index) [14] is based on the number of citations to specific articles and represents the impact and productivity of the individual researcher. While we could have attempted to look at h-indices from authors from various countries as another measure, this has its limitations, given that currently there are no estimates for what a “typical” or “average” h-index is for someone conducting research within medical education would be. h-indices are only relevant when determined in the context of a specific discipline [15]. As such, quantifying a specific h-index range for medical education researchers is needed to establish norms and conduct comparative analysis between countries.

### Key future research needed

There are number of critical questions raised by our paper. Why and how are countries with a relatively few number of medical schools, such as the Netherlands and New Zealand, so productive in medical education literature compared to countries with many more medical schools? Similarly the question must be asked as to why certain countries with more medical schools have low relative productivity with regards to medical education research. If it is true that some schools and countries focus their medical schools on education as opposed to research, what are the reasons for this? Is this due to high demand for physicians in these countries, financial (i.e. tuition) incentives for universities to promote education over research, or lack of funding for research? Our study raises these diverse and varied questions which could be the subject of future research medical education.

## Conclusions

Our analysis suggests a relatively small number of authors, networks and countries contribute most significantly the medical education research literature. While this helps to identify the main sources and arguably, drivers of medical education research publications, this must be interpreted with a critical awareness that our sample may be biased towards a mainly Western view of medical education research. Certainly, our finidings would suggest that we should strive for medical education research to become more prominent in low and middle income countries.

## Additional file

Additional file 1:
**Searches.**
