# Supplementary material for: Productivity in medical education research: an examination of countries of origin
Source: BMC Med Educ. 2014 Nov 18;14:243. doi: 10.1186/s12909-014-0243-8 (PMC4239316; doi:10.1186/s12909-014-0243-8)
Supplement: Additional file 1: — Searches. [file 12909_2014_243_MOESM1_ESM.doc]

## Additional file 1: Searches

### Evaluative studies of Medical Education

"education, medical"[MeSH Terms] AND (Clinical Trial[ptyp] OR Evaluation Studies[ptyp] or qualitative[Title/Abstract] OR themes[Title/Abstract])

### Other Medical Education Publications

"education, medical"[MeSH Terms] NOT (Clinical Trial[ptyp] OR Evaluation Studies[ptyp] or qualitative[Title/Abstract] OR themes[Title/Abstract])

### Searches for the Construction of Country Networks

USA

"education, medical"[MeSH Terms] AND (Clinical Trial[ptyp] OR Evaluation Studies[ptyp] or qualitative[Title/Abstract] OR themes[Title/Abstract]) USA[geo]

2321 hits February 8, 2011 and 2,953 hits April 7, 2014 (corrected to 2,927 post-1974 publications)

UK

"education, medical"[MeSH Terms] AND (Clinical Trial[ptyp] OR Evaluation Studies[ptyp] or qualitative[Title/Abstract] OR themes[Title/Abstract]) "United Kingdom"[geo]

679 hits February 8, 2011 and 857 hits April 7, 2014 (corrected to 851 post-1974 publications)

Canada

"education, medical"[MeSH Terms] AND (Clinical Trial[ptyp] OR Evaluation Studies[ptyp] or qualitative[Title/Abstract] OR themes[Title/Abstract]) Canada[geo]

440 hits February 8, 2011 and 639 hits April 7, 2014 (corrected to 638 post-1974 publications)

Australia

"education, medical"[MeSH Terms] AND (Clinical Trial[ptyp] OR Evaluation Studies[ptyp] or qualitative[Title/Abstract] OR themes[Title/Abstract]) Australia[geo]

204 hits February 8, 2011 and 282 hits April 7, 2014 (no pre-1975 publications)

Germany

"education, medical"[MeSH Terms] AND (Clinical Trial[ptyp] OR Evaluation Studies[ptyp] or qualitative[Title/Abstract] OR themes[Title/Abstract])] Germany[geo]

147 hits Feb 8, 2011 and 238 hits April 7, 2014 (no pre-1975 publications)

Netherlands

"education, medical"[MeSH Terms] AND (Clinical Trial[ptyp] OR Evaluation Studies[ptyp] or qualitative[Title/Abstract] OR themes[Title/Abstract]) Netherlands[geo]

155 hits February 8, 2011 and 226 hits April 7, 2014 (no pre-1975 publications)

Spain

"education, medical"[MeSH Terms] AND (Clinical Trial[ptyp] OR Evaluation Studies[ptyp] or qualitative[Title/Abstract] OR themes[Title/Abstract])] Spain[geo]

71 hits Feb 8, 2011 and 93 hits April 7, 2014 (no pre-1975 publications)

France

"education, medical"[MeSH Terms] AND (Clinical Trial[ptyp] OR Evaluation Studies[ptyp] or qualitative[Title/Abstract] OR themes[Title/Abstract])] France[geo]

65 hits Feb 8, 2011 and 93 hits April 7, 2014 (no pre-1975 publications)

Denmark

"education, medical"[MeSH Terms] AND (Clinical Trial[ptyp] OR Evaluation Studies[ptyp] or qualitative[Title/Abstract] OR themes[Title/Abstract])] Denmark[geo]

56 hits Feb 8, 2011 and 74 hits April 7, 2014 (no pre-1975 publications)

Sweden

"education, medical"[MeSH Terms] AND (Clinical Trial[ptyp] OR Evaluation Studies[ptyp] or qualitative[Title/Abstract] OR themes[Title/Abstract])] Sweden[geo]

59 hits Feb 8, 2011 and 72 hits April 7, 2014 (corrected to 71 post-1974 publications)

## Additional file 1 : Searches

### Evaluative studies of Medical Education

"education, medical"[MeSH Terms] AND (Clinical Trial[ptyp] OR Evaluation Studies[ptyp] or qualitative[Title/Abstract] OR themes[Title/Abstract])

### Other Medical Education Publications

"education, medical"[MeSH Terms] NOT (Clinical Trial[ptyp] OR Evaluation Studies[ptyp] or qualitative[Title/Abstract] OR themes[Title/Abstract])

### Searches for the Construction of Country Networks

USA

"education, medical"[MeSH Terms] AND (Clinical Trial[ptyp] OR Evaluation Studies[ptyp] or qualitative[Title/Abstract] OR themes[Title/Abstract]) USA[geo]

2321 hits February 8, 2011 and 2,953 hits April 7, 2014 (corrected to 2,927 post-1974 publications)

UK

"education, medical"[MeSH Terms] AND (Clinical Trial[ptyp] OR Evaluation Studies[ptyp] or qualitative[Title/Abstract] OR themes[Title/Abstract]) "United Kingdom"[geo]

679 hits February 8, 2011 and 857 hits April 7, 2014 (corrected to 851 post-1974 publications)

Canada

"education, medical"[MeSH Terms] AND (Clinical Trial[ptyp] OR Evaluation Studies[ptyp] or qualitative[Title/Abstract] OR themes[Title/Abstract]) Canada[geo]

440 hits February 8, 2011 and 639 hits April 7, 2014 (corrected to 638 post-1974 publications)

Australia

"education, medical"[MeSH Terms] AND (Clinical Trial[ptyp] OR Evaluation Studies[ptyp] or qualitative[Title/Abstract] OR themes[Title/Abstract]) Australia[geo]

204 hits February 8, 2011 and 282 hits April 7, 2014 (no pre-1975 publications)

Germany

"education, medical"[MeSH Terms] AND (Clinical Trial[ptyp] OR Evaluation Studies[ptyp] or qualitative[Title/Abstract] OR themes[Title/Abstract])] Germany[geo]

147 hits Feb 8, 2011 and 238 hits April 7, 2014 (no pre-1975 publications)

Netherlands

"education, medical"[MeSH Terms] AND (Clinical Trial[ptyp] OR Evaluation Studies[ptyp] or qualitative[Title/Abstract] OR themes[Title/Abstract]) Netherlands[geo]

155 hits February 8, 2011 and 226 hits April 7, 2014 (no pre-1975 publications)

Spain

"education, medical"[MeSH Terms] AND (Clinical Trial[ptyp] OR Evaluation Studies[ptyp] or qualitative[Title/Abstract] OR themes[Title/Abstract])] Spain[geo]

71 hits Feb 8, 2011 and 93 hits April 7, 2014 (no pre-1975 publications)

France

"education, medical"[MeSH Terms] AND (Clinical Trial[ptyp] OR Evaluation Studies[ptyp] or qualitative[Title/Abstract] OR themes[Title/Abstract])] France[geo]

65 hits Feb 8, 2011 and 93 hits April 7, 2014 (no pre-1975 publications)

Denmark

"education, medical"[MeSH Terms] AND (Clinical Trial[ptyp] OR Evaluation Studies[ptyp] or qualitative[Title/Abstract] OR themes[Title/Abstract])] Denmark[geo]

56 hits Feb 8, 2011 and 74 hits April 7, 2014 (no pre-1975 publications)

Sweden

"education, medical"[MeSH Terms] AND (Clinical Trial[ptyp] OR Evaluation Studies[ptyp] or qualitative[Title/Abstract] OR themes[Title/Abstract])] Sweden[geo]

59 hits Feb 8, 2011 and 72 hits April 7, 2014 (corrected to 71 post-1974 publications)
